# Excretion of Vancomycin-Resistant Enterococci by Wild Mammals

**DOI:** 10.3201/eid0806.010247

**Published:** 2002-06

**Authors:** David J. P. Mallon, John E. Corkill, Sarah M. Hazel, J. Sian Wilson, Nigel P. French, Malcolm Bennett, C. Anthony Hart

**Affiliations:** * University of Liverpool, Liverpool, United Kingdom

**Keywords:** Enterococci, vancomycin resistance, wild mammals, excretion

## Abstract

A survey of fecal samples found enterococcal excretion in 82% of 388 bank voles (Clethrionomys glareolus), 92% of 131 woodmice (Apodemus sylvaticus), and 75% of 165 badgers (Meles meles). Vancomycin-resistant enterococci, all Enterococcus faecium of vanA genotype, were excreted by 4.6% of the woodmice and 1.2% of the badgers, but by none of the bank voles.

Over the last decade, enterococci have emerged as a major cause of nosocomial infections, ranging from urinary tract and wound infections to life-threatening bacteremia (*1**–**3*). Enterococci are well suited as nosocomial pathogens because they readily colonize skin and mucous membranes, survive well in the environment, tolerate temperatures from 10°C to 45°C, survive in acid and alkaline conditions, and are intrinsically resistant to many antimicrobial drugs such as cephalosporins, fluoroquinolones, and aminoglycosides. The importance of this pathogen has been heightened by the emergence of multidrug-resistant enterococci, which has raised the specter of untreatable infections (*2**,**4*). Although a number of Enterococcus species exist, most human infections are caused by E. faecalis and E. faecium. E. faecium is isolated more frequently in Europe and E. faecalis in the United States (*1**,**2**,**5**,**6*). Among several phenotypes of glycopeptide resistance, VanA (teicoplanin and vancomycin resistance) and VanB (vancomycin resistance only) account for most resistant isolates (*1*). The genes encoding both VanA and VanB are transferable, inducible, and detectable in both E. faecalis and E. faecium (*7*).

We have recently shown that wild rodents can be a reservoir of antibiotic-resistant gram-negative bacteria (*8*). We now present data on carriage of vancomycin-resistant enterococci (VRE) by wild mammals.

## The Study

From 1997 to 2000, fecal samples were obtained from woodmice (Apodemus sylvaticus) and bank voles (Clethrionomys glareolus) in woodland and on small islands in a lake near farmland in the Wirral, northwest England. We collected 177 fecal samples from bank voles and 13 from woodmice captured on the islands and 211 from bank voles and 118 from woodmice captured in the woodland. Fecal samples from badgers (Meles meles) were obtained near their burrows in a 10X10-km area in Cheshire, England. The 165 samples from the badgers were collected in 2000. Fecal samples were stored frozen at -70°C until analyzed.

Isolation was attempted by using both enterococcosel agar and broth, neither incorporating vancomycin (BBL Microbiology Systems, Cockeysville, MD). On thawing, fecal samples were emulsified in an equal volume of sterile saline, and 10 μL was spread onto enterococcosel agar and incubated at 37°C for 48 hours. In addition, approximately 500 μL of each sample was added into enterococcosel broth and incubated at 37°C for 48 hours, after which samples were subcultured onto blood agar plates with a vancomycin disk (5 μg) on the main inoculum. Suspect colonies (esculin positive or from around the vancomycin disk) were further tested to confirm their identity as enterococci (Streptococcal Grouping Kit: Oxoid, Basingstoke, UK; API 20 Strep: Biomerieux, Basingstoke, UK). Provisionally identified VRE were tested for MICs of vancomycin and teicoplanin (E-test, AB BioDisk, Solna, Sweden).

Whole bacterial cell DNA was extracted by suspending 1 μL of pure culture in 250 μL of Chelex-100 (Bio-Rad Laboratories, Hercules, CA) in water slurry (5% wt/vol) and boiling for 10 minutes. Cell debris was removed by centrifugation (13,000 rpm for 15 minutes) and DNA extract stored at -70°C. Genetic identification of enterococci to species level and detection of vanA, vanB, vanC1, or vanC2/C3 and vanD ligase genes were performed by using a multiplex polymerase chain reaction amplification (*7*). This amplification detects both structural genes encoding D-alanine—D-alanine ligases and glycopeptide resistance genes. As controls, strains of E. faecalis (NCTC 775) and E. faecium (NCTC 12202) and previously characterized strains of E. faecium encoding vanA and vanB were included. The primers used (*7**,**9*) were VANAA1: 5´-GGG AAA ACG ACA ATT GC-3´; VANAA2: 5´-GTA CAA TGC GGC CGT TA-3´; VANBB1: 5´-ATG GGA AGC CGA TAG TC-3´; VANBB2: 5´-GAT TTC GTT CCT CGA CC-3´; VANC1/C1: 5´-GGT ATC AAG GAA ACC TC-3´; VANC1/C2: 5´-CTT CCG CCA TCA TAG CT-3´; VANC23/C1: 5´-CTC CTA CGA TTC TCT TG-3´; VANC23/C2: 5´-CGA GCA AGA CCT TTA AG-3´; VANDD1: 5´-TAA GGC GCT TGC ATA TAC CG-3´; VANDD2: 5´-TGC AGC CAA GTA TCC GGT AA-3´; ddl FAECALIS-E1: 5´-ATC AAG TAC AGT TAG TCTT-3´; ddl FAECALIS-E2: 5´-ACG ATT CAA AGC TAA CTG-3´; Ddl FAECIUM-F1: 5´-GCA AGG CTT CTT AGA GA-3´; and Ddl FAECIUM-F2: 5´-CAT CGT GTA AGC TAA CTTC-3´.

Amplicons were separated by electrophoresis through agarose (1% wt/vol) gels (Sigma-Aldrich, Poole, UK). Gels were stained in ethidium bromide, viewed by UV transillumination, and photographed.

Pulsed-field gel electrophoresis (PFGE) of macrorestricted chromosomal DNA was performed to establish the genetic relatedness of the VRE isolates (*10*). Bacteria were harvested from solid media and embedded in lysozyme (25 mg/mL)–agarose blocks. Released nucleases were neutralized with 25 mg/mL proteinase K (Sigma-Aldrich) at 50°C overnight. Chromosomal DNA was digested with Smal (40 U/block); gel slices were embedded in 1% (wt/vol) PFGE agarose gels (Bio-Rad Laboratories) and separated by electrophoresis on a CHEF 3D system (Bio-Rad Laboratories) in X 0.5 Tris-Borate-EDTA buffer. Electrophoresis conditions were an initial switch time of 1 second with a linear increase to 20 seconds after 20 hours at a buffer temperature of 14°C, 6 V/cm with a pulsing angle of 120°C (*10*).

Enterococcus spp. were detected in feces from 10 (76.9%) of 13 woodmice and 135 (76.3%) of 177 bank voles on the islands and 110 (93.2%) of 118 woodmice and 185 (87.7%) of 211 bank voles from the woodland site. A total of 123 (74.5%) of badger samples contained enterococci.

VRE were isolated from 6 (5.1%) of 118 woodmice samples from the woodland area only. Two (1.2%) of the badger samples yielded VRE. None of the bank voles at either site were excreting VRE. Seven of the eight VRE were obtained on direct culture on enterococcosel agar. The remaining VRE (from a woodmouse) was obtained from broth culture, as were each of the other VRE also isolated by direct culture. Each of the VREs had vancomycin MICs >64 mg/L and teicoplanin MICs 0.75–6.0 mg/L (Table). Biochemical identification with the API 20 Strep did not provide accurate species assignment. Each VRE had the vanA gene but none of the other resistance genes, and each was confirmed as E. faecium by polymerase chain reaction (Figure). PFGE analysis showed that six of the isolates had band patterns differing by >10 bands, but two woodmice isolates were closely related, differing by only two bands.

**Table:** Isolates of vancomycin-resistant enterococci from wild mammalsa

Isolate identification no.	Vancomycin MIC (mg/L)	Teicoplanin MIC (mg/L)
WMb 75	64	2.0
WM 123c	>256	1.5–2.0
WM 132	>256	6.0
WM 150	>256	0.75
WM 227c	>256	1.5
WM 242	>256	2.0
B 5022	>256	3
B5024	96	3

^a^All isolates in this table are Enterococcus faecium, vanA ge ne (polymerase chain reaction). ^b^Abbreviations used: WM, woodmouse, B, badger. ^c^Genetically similar by pulsed-field gel electrophoresis of macrorestricted chromosomal DNA (differing by two bands).

**Figure:**
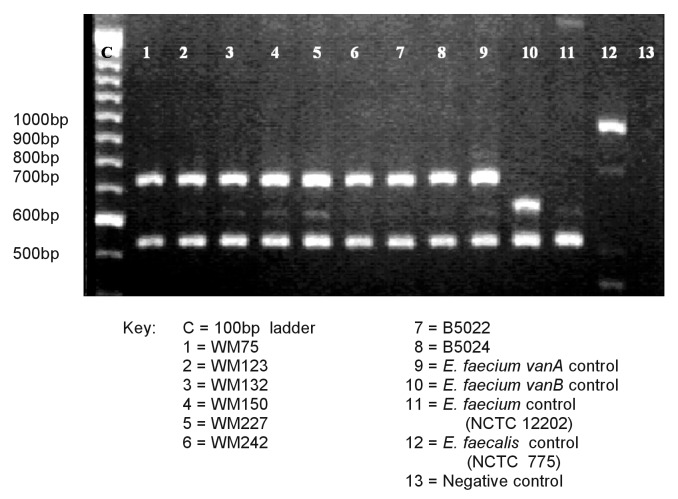
Polymerase chain reaction analysis of vancomycin-resistant enterococci isolates for glycopeptide resistance genotypes and species identification.

## Conclusions

This first study of enterococcal and specifically VRE excretion by wild mammals showed that enterococci appear to be part of the normal flora of most badgers (74.5%), woodmice (92%), and bank voles (82%), like many other animal species (*1*). We also found VRE in a small number of woodmice (5.1%) and badgers (1.2%). Each of our isolates was E. faecium, and each encoded the vanA but not vanB, vanC, or vanD genes. Although all had MICs for vancomycin >64 mg/L, only one had an MIC for teicoplanin of 6 mg/L, which does not correspond to the generally accepted VanA phenotype. However, an isolate of E. raffinosus has been described with a VanB phenotype yet vanA genotype (*11*). On PFGE of macrorestricted chromosomal DNA, only two isolates (both from woodmice) were genotypically related. The remaining six appeared unrelated. The two related isolates were from different woodmice (tagged on capture with transponders), trapped 6 months apart.

How the mammals acquired VRE and whether they are long-term carriers are less clear. The woodmice and bank voles may have been exposed in the woodland to either avoparcin (a glycopeptide antibiotic related to vancomycin) or fecal material from farm animals; however, avoparcin had not been used for any livestock raised in proximity to the sites, and the samples (including the badger samples) were taken after the use of avoparcin as a growth promoter had been banned. None of the bank voles were excreting VRE, in spite of the fact that they can be reservoirs for enterococci. In general, bank voles tend to be herbivorous and have a limited territory, beyond which they rarely stray. Woodmice and badgers are omnivorous and will travel distances in search of food and territory (*12*).

In conclusion, we demonstrated that woodmice and badgers can excrete VRE in their feces and may be an unexpected reservoir for such bacteria. However, bank voles occupying the same habitat as the woodmice did not excrete VRE. How long such reservoirs may persist and if the bacteria can be transmitted to other animals, including humans, remain to be determined.
